# Ruptured Splenic Ectopic Pregnancy: The Importance of Considering Nontubal Sites

**DOI:** 10.1155/crog/8867392

**Published:** 2025-08-06

**Authors:** Yomna Fahmy, Claire Ross, Nora Kiss, Duaa Gumaa, Ee Xuan Ngeyu, Despina Mavridou, Rasana Bajracharya

**Affiliations:** ^1^Department of Obstetrics and Gynaecology, Maidstone and Tunbridge Wells NHS Trust, Tunbridge Wells, UK; ^2^Department of Surgery, Maidstone and Tunbridge Wells NHS Trust, Tunbridge Wells, UK

## Abstract

**Objective:** Splenic ectopic pregnancy is an exceptionally rare and life-threatening form of abdominal ectopic pregnancy, often presenting significant diagnostic and management challenges. We report a case of ruptured splenic ectopic pregnancy initially suspected to be a tubal miscarriage.

**Case Report:** A 36-year-old woman presented with mild left-sided pelvic pain, no vaginal bleeding and a positive pregnancy test. This was an unplanned pregnancy, and she was not sure of her LMP. This presentation in early pregnancy requires ruling out an ectopic pregnancy through clinical assessment and laboratory investigations. Clinical examination showed normal observations and generalized abdominal tenderness with no signs of peritonism. Initial investigations revealed a significantly elevated *β*-hCG of 24,076 IU/L, and transvaginal ultrasound showed an empty uterus. Given the findings, an ectopic pregnancy was suspected, and diagnostic laparoscopy was performed. During laparoscopy, no ectopic pregnancy was identified in the pelvis, both tubes looked normal, but a left fimbrial cyst was noted, and 300 mL of haemoperitoneum was observed, without active bleeding. A tubal miscarriage was presumed with the plan to follow up *β*-hCG to confirm resolution of the pregnancy. Postoperatively, the patient developed worsening pain and a significant haemoglobin drop to 97 g/L, inconsistent with the amount of intraoperative blood loss which raised the suspicion of extrapelvic ectopic pregnancy with active bleeding. A contrast-enhanced CT scan identified free blood surrounding the spleen and a 25-mm peripherally enhancing lesion with venous drainage into the splenic vein, consistent with a ruptured splenic ectopic pregnancy. An urgent multidisciplinary team discussion led to surgical management via midline laparotomy and splenectomy. The postoperative course was complicated by ileus, which resolved with conservative management. The patient's *β*-hCG levels progressively declined, confirming resolution.

**Conclusion:** This case highlights the importance of considering splenic ectopic pregnancy when *β*-hCG is markedly elevated, and no pelvic ectopic pregnancy is identified. Prompt imaging and diagnosis are crucial to prevent morbidity associated with delayed management.

## 1. Introduction

Ectopic pregnancy occurs in approximately 1%–2% of all pregnancies, with the majority implanting in the fallopian tubes [1], whilst abdominal ectopic pregnancy occurs in approximately 1.2%–1.4% of all reported pregnancies worldwide [2]. In the United Kingdom, nearly 12,000 ectopic pregnancies are diagnosed each year, which gives a prevalence of 1.1% [3]. Abdominal ectopic pregnancies, particularly splenic ectopic pregnancies, are exceedingly rare and pose significant diagnostic and therapeutic challenges. Given the nonspecific clinical presentation, misdiagnosis is common, often leading to delayed treatment and increased morbidity. This report presents a case of a ruptured splenic ectopic pregnancy initially misdiagnosed as a tubal miscarriage, emphasizing the need for heightened clinical suspicion and appropriate imaging.

## 2. Case Presentation

A 36-year-old multiparous woman presented to the Early Pregnancy Assessment Unit with mild left-sided pelvic pain with a positive pregnancy test and no vaginal bleeding. She was a Para 3 with previous uncomplicated pregnancies and had normal vaginal deliveries 16, 13 and 12 years ago. This was an unplanned pregnancy; she was not using contraception and was not sure of her LMP. She is of White British background and had no significant past medical history, and her past surgical history included an appendectomy but no other surgeries. She was allergic to penicillin and does not drink alcohol, and she uses vapes. Clinical examination revealed normal observations with BP 102/61, HR 79, Temp 36.8 and RR 16; abdominal examination showed generalized lower abdominal tenderness with no guarding, rigidity or rebound tenderness. Initial laboratory investigations showed an elevated *β*-hCG of 24,076 IU/L, progesterone 14 and a haemoglobin level of 130 g/L with normal liver and kidney functions. Transvaginal ultrasound revealed an empty uterus with an endometrial thickness of 19.6 mm and a fluid-filled 20 × 14 mm area in the left adnexa, with no free fluid in the pouch of Douglas (Figures S1 and S2).

Given the elevated *β*-hCG and absence of an intrauterine pregnancy, an ectopic pregnancy was suspected. Diagnostic laparoscopy was performed, revealing a left fimbrial cyst; both fallopian tubes looked normal with 300 mL of haemoperitoneum, but no identifiable ectopic pregnancy in the pelvis or a source of active bleeding. The abdomen was inspected, with no abnormalities noted. Based on these findings, a tubal miscarriage was presumed with a plan to repeat *β*-hCG to confirm resolution of the pregnancy. The fimbrial cyst was excised and sent for histopathology.

Postoperatively, the patient developed worsening pain, and her haemoglobin dropped to 97 g/L. This significant decline was inconsistent with intraoperative blood loss, raising concern for ongoing haemorrhage and a possible missed extrapelvic ectopic pregnancy. An evolving intrauterine pregnancy must be excluded, and with *β*-hCG above the discriminatory level of 300–1000 mIU/mL, an evolving intrauterine pregnancy should be visible on transvaginal scan [4] thus the need to consider extrapelvic implantation. A contrast-enhanced CT scan revealed free blood surrounding the spleen and a 25-mm peripherally enhancing lesion within the spleen, with venous drainage into the splenic vein, consistent with a ruptured splenic ectopic pregnancy (Table 1; Figures S3 and S4).

A multidisciplinary team, including gynaecology, general surgeons and interventional radiologists, was consulted and decided that embolization would not be suitable in this case and urgent surgical intervention was planned. The patient underwent a midline laparotomy where 2 L of blood was present in the pelvis, the ectopic pregnancy was identified in the spleen and total splenectomy was performed. She required four units of blood transfusion and was admitted to ITU. Postoperatively, she developed ileus, which was managed conservatively with an NG tube and glycerol suppositories. Serial *β*-hCG levels demonstrated a progressive decline, confirming the resolution of the ectopic pregnancy.

Histopathology confirmed the excision of a benign, simple serous fimbrial cyst with no trophoblasts or chorionic villi. The spleen examination confirmed the presence of chorionic villi and a placental site reaction confirming ectopic gestation (Figure S5).

## 3. Discussion

Most ectopic pregnancies implant in the fallopian tubes, with transvaginal ultrasound and diagnostic laparoscopy being the gold standard for evaluation [1]; however, these modalities primarily assess the pelvis and may fail to identify intra-abdominal or intraparenchymal ectopic pregnancies. Combined transvaginal ultrasonography and serial quantitative beta-hCG measurements are approximately 96% sensitive and 97% specific for diagnosing ectopic pregnancy [4]. In this case, the initial diagnostic laparoscopy did not reveal an ectopic pregnancy, leading to a presumed tubal miscarriage diagnosis. The persistently high *β*-hCG levels and worsening anaemia prompted further investigation, ultimately leading to the diagnosis of splenic ectopic pregnancy. It is important in such cases to consider other nontubal sites of ectopic pregnancy such as the cervix, ovary, abdomen, interstitial portion of the fallopian tube and caesarean scars. The incidence range of nontubal EP (NT-EP) is between 5% and 8.3% of all EP, and it has increased in the last two decades with the widespread use of assisted reproductive techniques (ARTs) [5].

Whilst abdominal pregnancies constitute approximately 1% of all pregnancies, mortality rates are 7.7 times higher than in tubal pregnancy and 89.8 times higher than in intrauterine pregnancy [6]. Studdiford's criteria to diagnose a primary abdominal pregnancy are normal bilateral fallopian tubes and ovaries, the absence of uteroperitoneal fistula and pregnancy related exclusively to the peritoneal surface and early enough to eliminate the possibility of secondary implantation following a primary location in the tube [7]. Splenic ectopic pregnancy is a rare entity with few reported cases in the literature. It can result from implantation following tubal abortion or direct fertilization within the spleen. The condition is highly vascular, predisposing patients to life-threatening haemorrhage upon rupture. Splenic ectopic pregnancy is easily missed and mostly diagnosed after substantial emergency bleeding, which is caused by an unsecure splenic pregnancy placenta, a weak gestational sac and the lack of protection of the myometrium [8]. Imaging modalities such as contrast-enhanced CT or MRI play a crucial role in detecting these ectopic pregnancies, especially when the initial laparoscopy is inconclusive; they are most reliable in describing the relationship between the location of the gestational sac and surrounding tissue and in assessing the risk of rupture and other complications [4, 8]. There is no consensus on the best management option for splenic ectopic pregnancies; there are cases reported in literature where patients were managed with minimally invasive procedures like angiography coil embolization [2], methotrexate or more commonly surgical excision either via laparoscopy or more commonly laparotomy, especially in cases with haemodynamic instability [9, 10].

Like previously reported cases, our patient presented with acute abdominal pain and haemodynamic instability, underscoring the life-threatening nature of splenic implantation. Similar to the case described by Eghbali et al. [1], the initial imaging failed to definitively localize the ectopic gestation, leading to diagnostic delays. However, unlike some earlier reports where diagnosis was established only intraoperatively or on histopathology, our case benefited from heightened clinical suspicion and timely laparoscopy, allowing for surgical intervention before complete exsanguination. In contrast to other cases where the spleen was preserved, our patient required splenectomy due to uncontrolled bleeding, emphasizing the variability in surgical outcomes. Unlike many reported patients, our subject had no known risk factors such as prior ectopic pregnancies or assisted reproductive technology use. This case thereby contributes to the spectrum of presentations and management strategies of splenic ectopic pregnancies and reinforces the importance of including upper abdominal sites in the differential when imaging is inconclusive.

## 4. Conclusion

This case underscores the importance of considering abdominal ectopic pregnancy, particularly splenic implantation, in patients with a markedly elevated *β*-hCG and negative findings on pelvic ultrasound and laparoscopy. Early recognition and appropriate imaging are critical for timely diagnosis and management, preventing significant morbidity and potential mortality.

## 5. Limitations

This report describes a single, rare case of splenic ectopic pregnancy, which limits the generalizability of its findings. As a case report, it does not allow for broader conclusions regarding causality, risk factors or treatment efficacy. Additionally, due to the urgent nature of the presentation, there were constraints on preoperative imaging and diagnostic steps, which may have limited a more comprehensive evaluation of the ectopic site before surgery. Furthermore, the rarity of splenic ectopic pregnancies makes it difficult to compare standardized treatment protocols or outcomes. Finally, whilst we have included a brief comparison with other reported cases, the limited number of similar reports in the literature restricts the depth of analysis that can be performed.

## Figures and Tables

**Table 1:** Blood results at presentation and follow-up.

**At presentation**	**Postlaparoscopy**	**Day 2 postlaparotomy**	**Day 3 postlaparotomy**
Hb 130 g/L	Hb 97 g/L		
Plt 288 × 10^9^	Plt 235 × 10^9^		
WCC 6 × 10^9^	WCC 11 × 10^9^		
CRP 9 mg/L	CRP 8 mg/L		
Na 139 mmol/L			
K 3.9 mmol/L			
Alt 15 mmol/L			
Normal coagulation screen			
*β*-hCG 24,076 mIU/mL, progesterone 14 ng/mL	*β*-hCG 23,769 mIU/mL	*β*-hCG 5011 mIU/mL	*β*-hCG 2715 mIU/mL

## Data Availability

Data sharing is not applicable to this article as no new data were created or analyzed in this study.

## Supporting Information

Supporting Information 1 Figure S1: Transvaginal ultrasound showing an empty uterine cavity with endometrial thickness 19.6 mm.

Supporting Information 2 Figure S2: Transvaginal ultrasound showing a fimbrial cyst.

Supporting Information 3 Figure S3: Axial CT scan showing a splenic ectopic pregnancy (arrow) within the spleen.

Supporting Information 4 Figure S4: Coronal CT scan showing a splenic ectopic pregnancy (arrow) within the spleen.

Supporting Information 5 Figure S5: Gross specimen of the spleen after splenectomy, showing the ectopic pregnancy.
