# Supplementary material for: Ruptured Splenic Ectopic Pregnancy: The Importance of Considering Nontubal Sites
Source: Case Rep Obstet Gynecol. 2025 Aug 6;2025:8867392. doi: 10.1155/crog/8867392 (PMC12349982; doi:10.1155/crog/8867392)
Supplement: Supporting Information 1 — Figure S1: Transvaginal ultrasound showing an empty uterine cavity with endometrial thickness 19.6 mm. [file 8867392.f1.pdf]

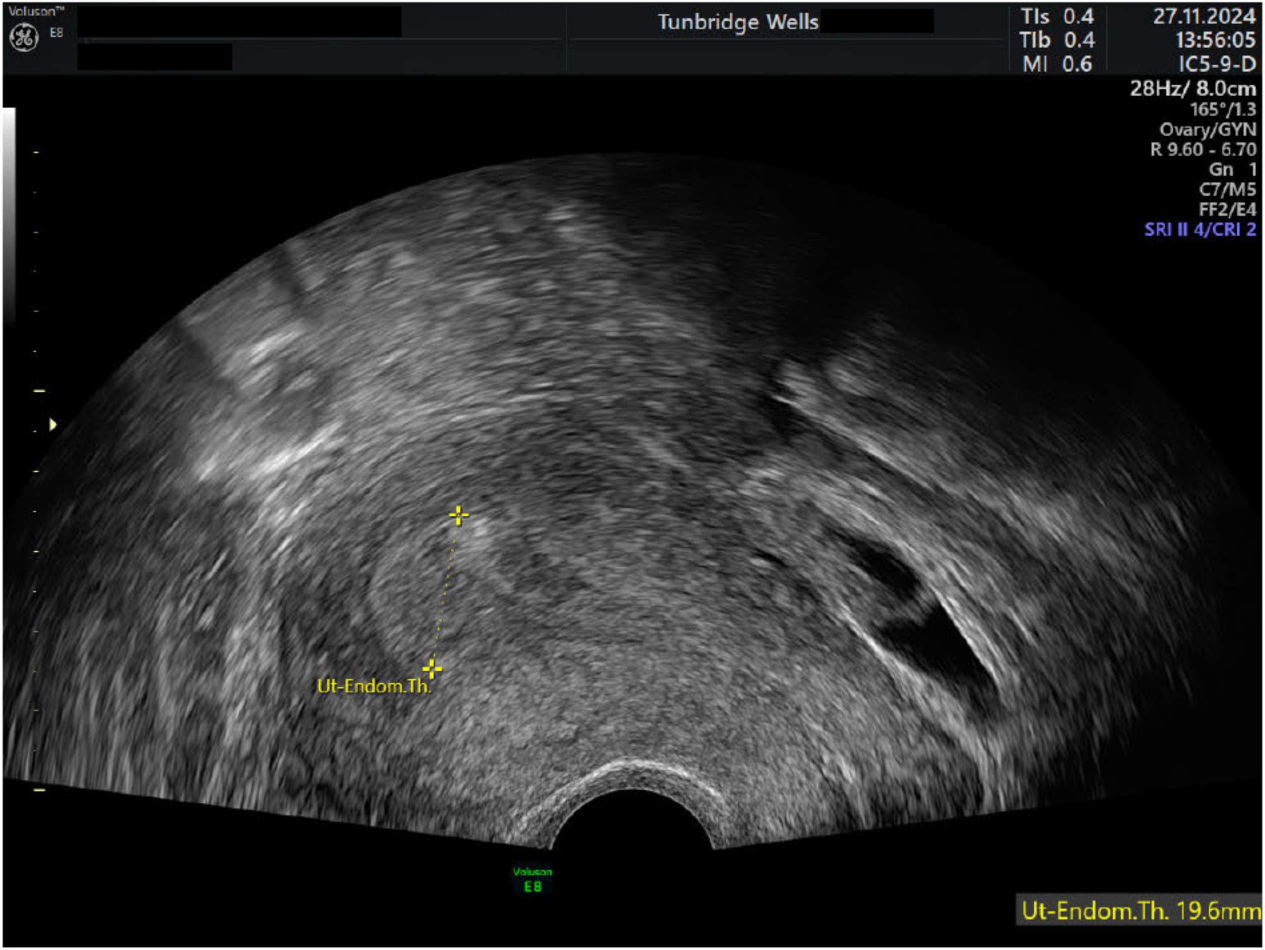
Voluson™
E8
[redacted]
[redacted]
Tunbridge Wells [redacted]
TIs 0.4
TIb 0.4
MI 0.6
27.11.2024
13:56:05
IC5-9-D
28Hz/ 8.0cm
165°/1.3
Ovary/GYN
R 9.60 - 6.70
Gn 1
C7/M5
FF2/E4
SRI II 4/CRI 2
Ut-Endom.Th.
Voluson
E8
Ut-Endom.Th. 19.6mm
