# Supplementary figures and images for: Ruptured Splenic Ectopic Pregnancy: The Importance of Considering Nontubal Sites
Source: Case Rep Obstet Gynecol. 2025 Aug 6;2025:8867392. doi: 10.1155/crog/8867392 (PMC12349982; doi:10.1155/crog/8867392)

? FIMBRIAL CYST? HYDROSALPINX

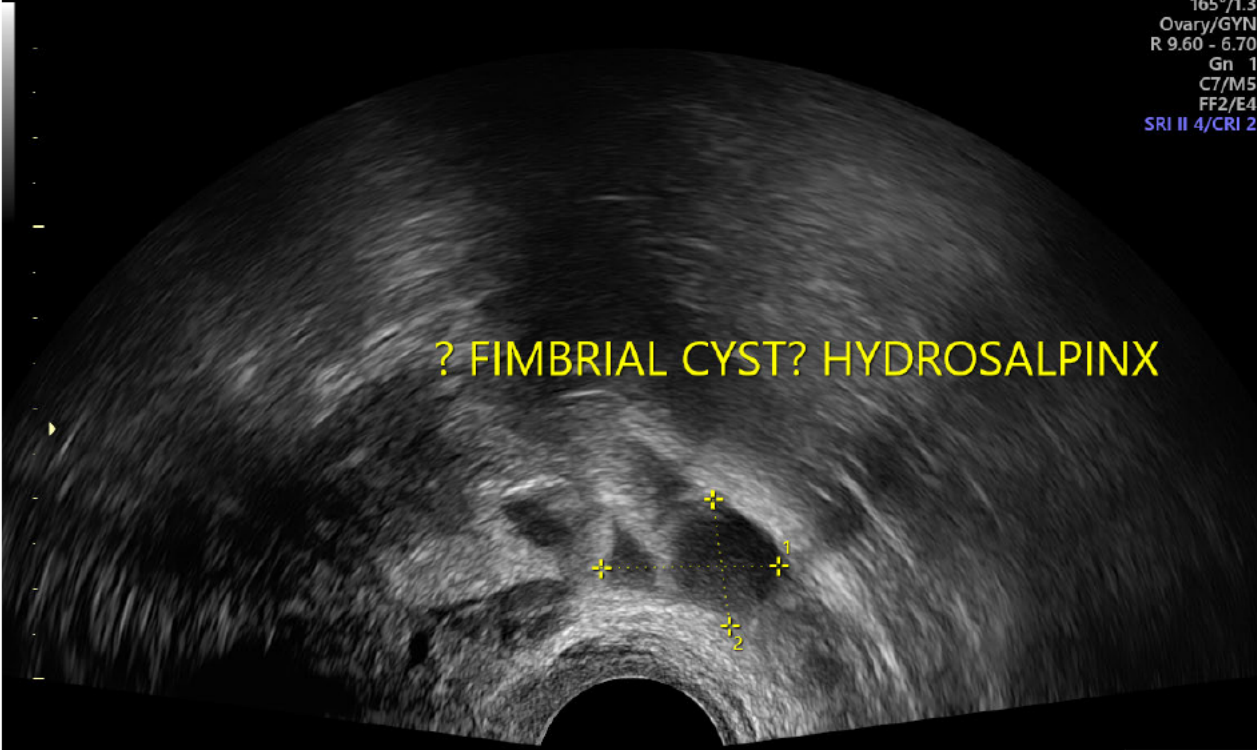

Supplement: Supporting Information 2 — Figure S2: Transvaginal ultrasound showing a fimbrial cyst. [file 8867392.f2.pdf]

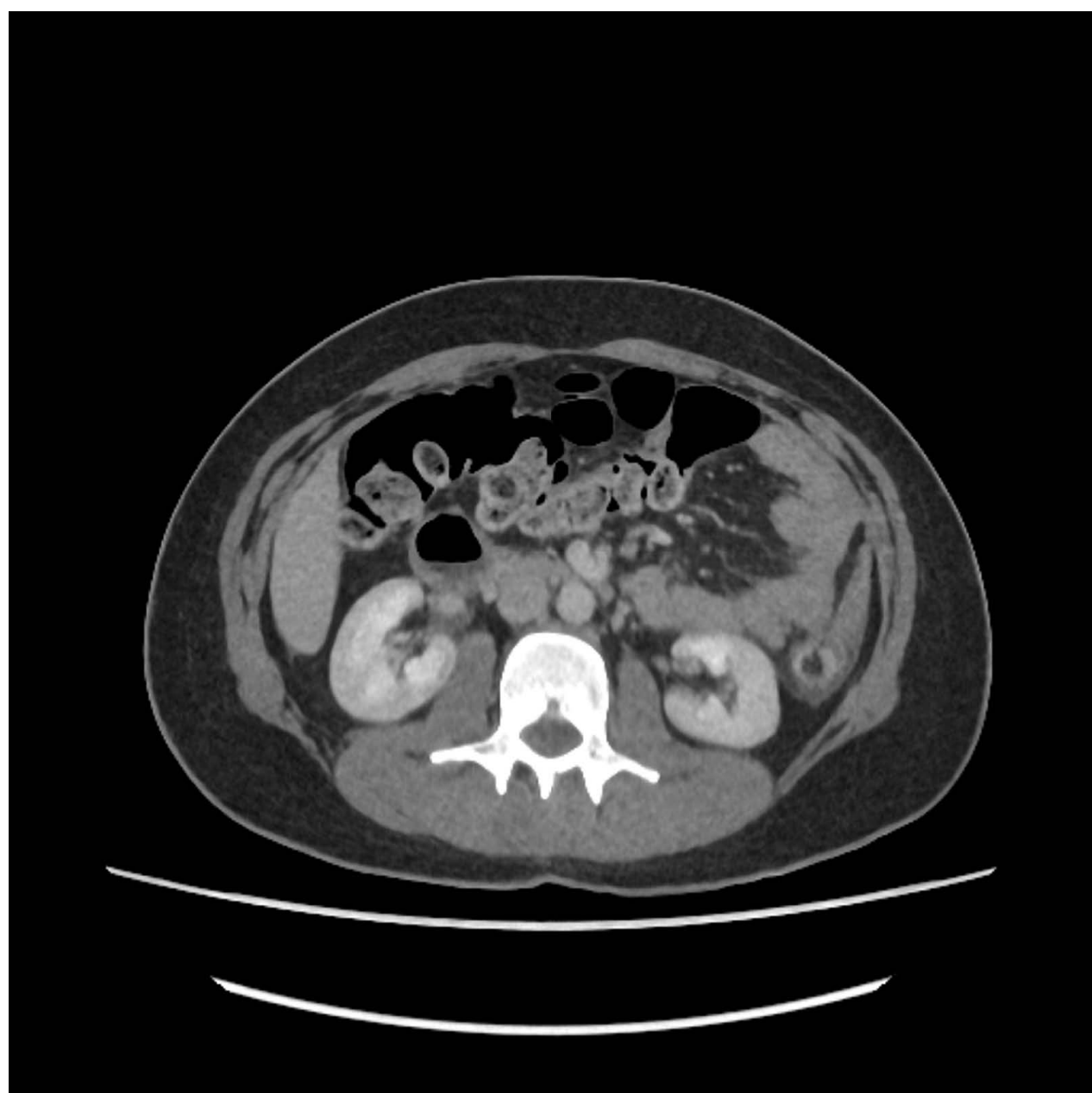

Supplement: Supporting Information 4 — Figure S4: Coronal CT scan showing a splenic ectopic pregnancy (arrow) within the spleen. [file 8867392.f4.pdf]

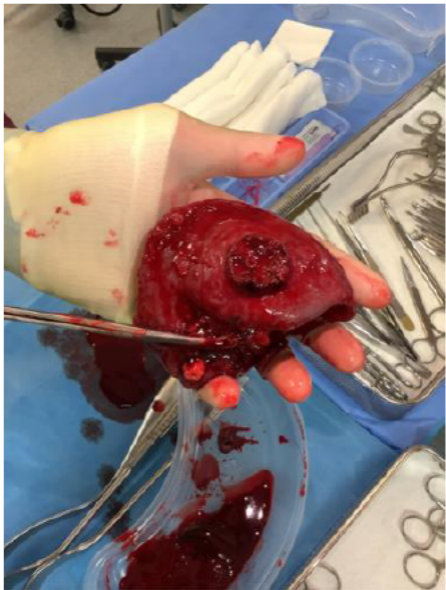

Supplement: Supporting Information 5 — Figure S5: Gross specimen of the spleen after splenectomy, showing the ectopic pregnancy. [file 8867392.f5.pdf]
