# Supplementary material for: Ruptured Splenic Ectopic Pregnancy: The Importance of Considering Nontubal Sites
Source: Case Rep Obstet Gynecol. 2025 Aug 6;2025:8867392. doi: 10.1155/crog/8867392 (PMC12349982; doi:10.1155/crog/8867392)

**Supplementary Figure 3.** Axial CT scan showing a splenic ectopic pregnancy (arrow) within the spleen.

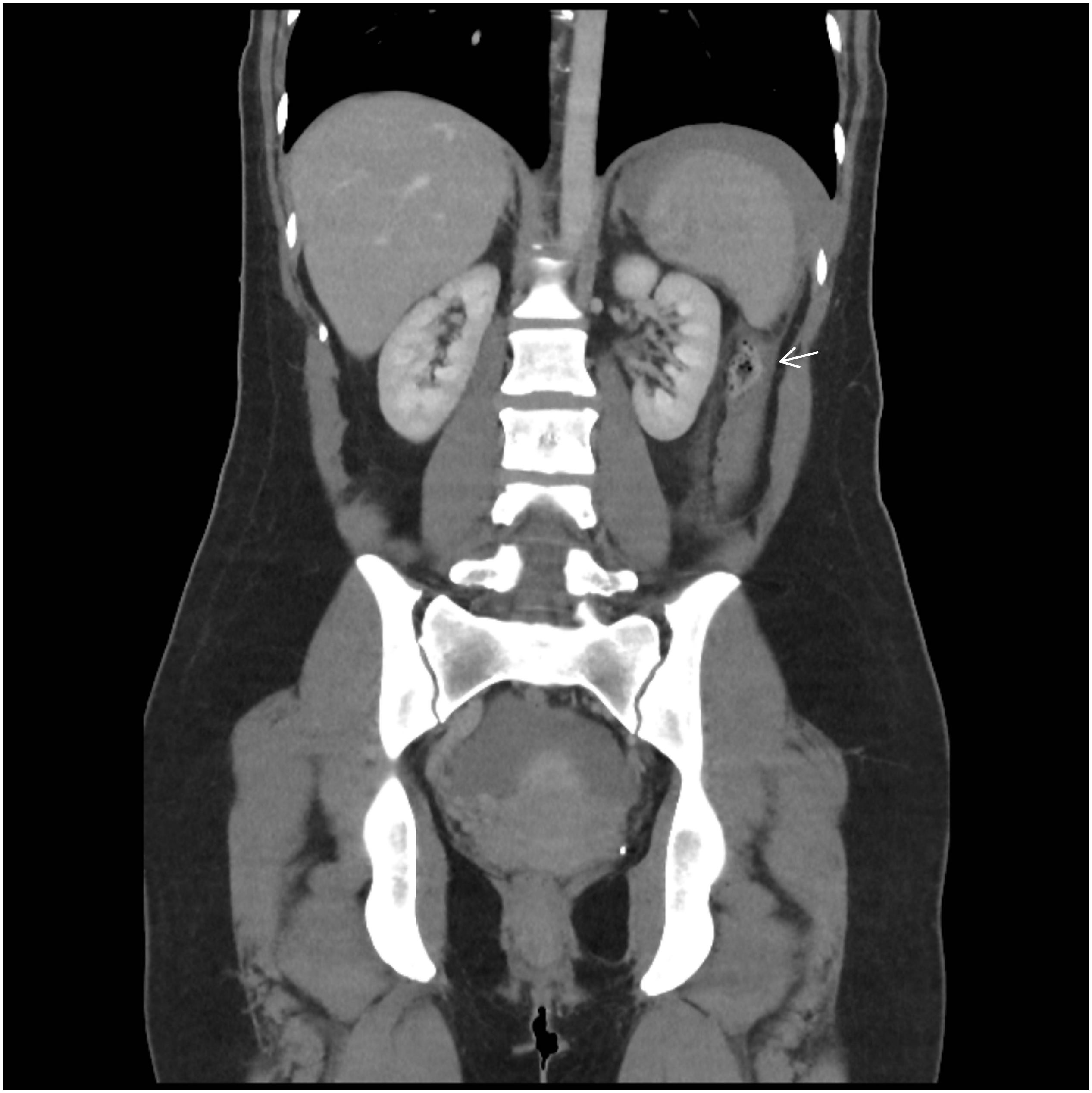

Supplement: Supporting Information 3 — Figure S3: Axial CT scan showing a splenic ectopic pregnancy (arrow) within the spleen. [file 8867392.f3.pdf]
